# Role of Intravenous Levetiracetam in Acute Seizure Management

**DOI:** 10.3389/fneur.2014.00109

**Published:** 2014-06-27

**Authors:** Batool Kirmani

**Affiliations:** ^1^Texas A&M HSC College of Medicine, Scott and White Healthcare, Temple, TX, USA

**Keywords:** levetiracetam, acute seizure management, anticonvulsants, treatment, epilepsy

Epilepsy is a chronic medical condition that still poses a challenge in terms of treatment. There are a variety of anticonvulsants available today, but these do not prevent frequent hospital admissions and emergency room visits. Benzodiazepines, however, remain the first line treatment in acute seizure management. The approval of phenytoin, fosphenytoin, intravenous valproate, and rectal diazepam has given more options to the physicians for acute management. The approval of intravenous levetiracetam has provided another option for physicians with patients who failed the other approved anticonvulsants. Intravenous levetiracetam is approved for patients 4 years and older as an alternative to oral treatment. There have been various case reports, case series, and retrospective studies showing the efficacy of intravenous levetiracetam both in status epilepticus (SE) and acute seizure exacerbation. These studies reported favorable response of intravenous levetiracetam in both adults and children. Data have even shown good results in neonates and preterm children. In this volume, we have included articles from renowned researchers in the field of neurology and epilepsy who have covered the various aspects of these agents in detail including the properties, mechanism of action, pharmacology, neurobehavioral effects, and the roles of these agents in special populations. These data further show that intravenous levetiracetam can be used in acute seizure management.

This book opens with a chapter by Jennifer L. DeWolfe and Jerzy P. Szaflarski (1) that discusses in detail the role of intravenous levetiracetam in the critical care setting. The literature review was conducted, which showed that intravenous levetiracetam is effective in terminating different types of seizures including SE, post-traumatic and tumor-related seizures, seizures due to stroke, and intraparenchymal hemorrhage. The pharmacokinetics of this agent in special populations including elderly, pregnant, and neurocritical patients is also discussed in this chapter. The authors concluded that there is still need for larger prospective trials, but based on current data, the drug appears to be safe and better tolerated in different subgroups of seizure population. The second chapter by Wright et al. (2) reviewed the current literature about the pharmacology and pharmacokinetics of intravenous levetiracetam and the safety profile of this drug in adults and children. The article also showed unique mechanism of action, linear pharmacokinetics, and no known drug–drug interactions with other anticonvulsants, which makes it a viable option for acute seizure management in both adults and children. The third chapter by Aceves et al. (3) further discusses in detail the current data regarding the safety and tolerability of intravenous levetiracetam in children and neonates in the management of SE and acute repetitive seizures. The authors also emphasize the need for a larger prospective multicenter trial to further define the roles of these anticonvulsants in this population subgroup.

Status epilepticus is a major neurological emergency that is associated with high morbidity and mortality. SE can cause significant neuronal injury and the patients who survive SE develop long term neurological sequelae including major cognitive issues. The fourth chapter is by Laxmikant S. Deshpande and Robert J. DeLorenzo who discuss the mechanisms of levetiracetam in the control of SE and epilepsy. (4) The authors in their review focused on the unique anticonvulsant properties of levetiracetam and concluded that the unconventional mechanism of action, favorable safety profile, and lack of sedating effects makes it a viable candidate to be used in the management of this neurological emergency. The fifth chapter by Hae Won Shin and Robin Davis is the review of levetiracetam as a first line treatment in SE in adult patients and the need for larger prospective trials in the future (5).

The sixth chapter by Fonkem et al. (6) and seventh chapter by Bernett et al. (7) focus on a special population subgroup – those with brain tumor-related seizures. Fonkem et al. (6) in his literature review article has shown that levetiracetam is an attractive option for brain tumor-related seizures because levetiracetam can increase the sensitivity of glioblastoma tumors to the chemotherapy drug temozolomide. Levetiracetam can also be used as prophylaxis in patients with brain tumors and in patients undergoing neurological surgery. Bernett et al. (7) summarize the limited data available, which show the potential risk of neurobehavioral side-effects with levetiracetam in brain tumor-related seizure patients and the need for future research.

The next four chapters focus on traumatic brain injury and the neuroprotective properties of Levetiracetam. The eighth chapter by Shetty et al. (8) discusses in detail the data available about the potential mechanism of epileptogenesis and neuroprotective properties of this agent, which are beneficial in treating seizures associated with neurological conditions like SE, stroke, and traumatic brain injury. The ninth chapter by Kovacs et al. (9) discusses the blast traumatic brain injury models, neuropathology, and implications for seizure risk. In this review, the authors reviewed the pathological results, which also included immunohistochemical and special staining approaches from recent preclinical explosive blast studies to better understand the mechanism by which explosions cause brain injury. This is followed by chapter by Kirmani et al. (10), which discusses the role of intravenous levetiracetam in severe traumatic brain injury patients. The authors concluded, based on current literature review, that intravenous levetiracetam can be considered as a viable option in acute care settings if phenytoin is unavailable or the administration is not feasible due to side-effects.

The next chapter by Benge et al. (11) summarizes the limited current data available about the neurobehavioral effects of levetiracetam in traumatic brain injury patients and the need for future studies.

The last chapter is an original article by Rogers et al. (12) in which effects of levetiracetam on the mitochondrial membrane potential of neuronal and non-neuronal cells were examined *in vitro* to determine if levetiracetam influences metabolic processes in these cell types. These results suggested that both neuronal and non-neuronal anticonvulsant properties of levetiracetam involve control over energy metabolism.

This book provides a comprehensive review of the role of intravenous levetiracetam in acute seizure management and emphasizes the need of larger prospective trials to further define the role of this anticonvulsant.

## Conflict of Interest Statement

The author declares that the research was conducted in the absence of any commercial or financial relationships that could be construed as a potential conflict of interest.
